# Blister-Actuated LIFT Printing for Multiparametric Functionalization of Paper-Like Biosensors

**DOI:** 10.3390/mi10040221

**Published:** 2019-03-28

**Authors:** Lars Hecht, Korbinian Rager, Martynas Davidonis, Patricia Weber, Günter Gauglitz, Andreas Dietzel

**Affiliations:** 1 Institute of Microtechnology (IMT), Technische Universität Braunschweig, Alte Salzdahlumer Str. 203, D-38124 Braunschweig, Germany; k.rager@tu-braunschweig.de (K.R.); mdavidonis@gmail.com (M.D.); 2 Institute for Physical and Theoretical Chemistry, University of Tübingen, Auf der Morgenstelle 18, D-72076 Tübingen, Germany; patricia.weber@uni-tuebingen.de (P.W.); guenter.gauglitz@uni-tuebingen.de (G.G.)

**Keywords:** multiparametric lateral flow test, liquid dispensing, laser induced forward transfer, microarray fabrication, paper-like bio sensors

## Abstract

Laser induced forward transfer (LIFT) is a flexible digital printing process for maskless, selective pattern transfer, which uses single laser pulses focused through a transparent carrier substrate onto a donor layer to eject a tiny volume of the donor material towards a receiver substrate. Here, we present an advanced method for the high-resolution micro printing of bio-active detection chemicals diluted in a viscous buffer solution by transferring droplets with precisely controllable volumes using blister-actuated LIFT (BA-LIFT). This variant of the LIFT process makes use of an intermediate polyimide layer partially ablated by the laser pulses. The expanding gaseous ablation products lead to blisters in the polyimide and ejection of droplets from the subjacent viscous solution layer. A relative movement of donor and receiver substrates for the transfer of partially overlapping pixels is realized with a custom-made positioning system. Using a specially developed donor ink containing bio-active components presented method allows to transfer droplets with well controllable volumes between 20 fL and 6 pL, which is far more precise than other methods like inkjet or contact printing. The usefulness of the process is demonstrated by locally functionalizing laser-structured nitrocellulose paper-like membranes to form a multiparametric lateral flow test. The recognition zones localized within parallel micro channels exhibit a well-defined and homogeneous color change free of coffee-ring patterns, which is of utmost importance for reliable optical readout in miniature multiparametric test systems.

## 1. Introduction

One of the most important steps in the fabrication of biosensors or microarrays is the functionalization or loading of selected surface areas by dispensing very precisely defined volumes of reaction chemicals in liquid form. Multiple techniques are known and well established for this surface functionalization step: For some laboratory experiments in which rather large droplet volumes and low reproducibility are acceptable manual application with dispensers is viable. However, in most cases, the molecules are printed or “spotted” in the reaction zones using commercially available micro-arrayers, which are able to dispend volumes in the range of pL to µL. These systems can be roughly divided into contact printing systems, which rely on pins that touch the target substrate and non-contact jetting systems, which form and propel droplets toward the target substrate, both being characterized by specific advantages and disadvantages [1].

The pin printing method, a well-established technique, uses a single rod or needle with a flat or concave shaped tip, which is dipped into a reagent reservoir where it captures a small amount of fluid. The droplet is then carried on the tip to the target area where it is transferred during contact between the pin and the substrate. For each subsequent droplet transfer the pin has to be dipped into the reservoir. Advantages of this method include very simple and fast washing steps in between different samples and a very controllable droplet formation. However, evaporation of the sample on the tip while moving the gantry to the substrate can be an issue with liquid volumes in the range of hundreds of picoliters [2]. An additional problem is the lack of volume control—depending on the receiver surface, the applied volume can vary and a precise dosage of reagents can be challenging. Furthermore, delicate substrates can locally be damaged by the contact, which could interfere with the biosensor readout. While an optimization of the throughput speed can be achieved by using an array of pins to transfer multiple droplets at once [3], the flexibility of this approach is very limited by the fixed pitch in between the pins.

Non-contact microarrayers use glass capillaries actuated with piezo membranes to eject and propel droplets toward the substrate. In modern microarrayers, the droplet volume can be precisely controlled and monitored during the jetting process with optical methods. Furthermore, easy washing steps and quick exchange of the spotting liquid by dipping the capillaries in an array of temperature-controlled wells can be realized. A disadvantage of this method is the limitation to a small range of sample viscosities, since the required pressure to eject very viscous samples through the small capillary orifices is too high. In a functionalized area created by a single droplet a coffee-ring pattern originates from the capillary flow induced by the differential evaporation rates across the drop. This pattern is detrimental because a uniform functionalization is providing the optimum for optical read out of a biosensor. Since droplet sizes of 50 pL are the current minimum for commercial spotters [4], a homogenization of the functionalization by printing patterns of multiple droplets becomes a problem when biosensors are further miniaturized. 

For larger scale applications the use of inkjet-printing is also feasible. There are a number of printheads and/printer systems available, which can deliver droplet volumes ranging from a few pL [5] up to several nL [6]. However, these systems are quite limited in their adaptability to new “inks” and susceptible to clogging when changing the print material. Ensuring that each nozzle is printing at any given moment remains a challenge. 

An alternative approach for non-contact transfer, which allows an unprecedented accuracy in dosage, is given by so called laser induced forward transfer (LIFT). It is a flexible digital printing process for mask-less, selective material and pattern transfer, which uses single laser pulses focused through a transparent carrier substrate on a donor layer to eject pixels of the material onto a receiver substrate. While it was first developed decades ago for the area-selective deposition of thin metal films [7], a number of modifications and variants of the basic LIFT principle have later been reported, which enable the transfer of numerous different materials in the solid or liquid phase [8,9]. 

Here, we present a novel method for the micro printing of bio-active detection chemicals diluted in a viscous buffer solution using blister-actuated LIFT (BA-LIFT). This variant of the LIFT process makes use of an intermediate polyimide layer partially ablated by the laser pulses [10]. The expanding gaseous ablation products lead to blisters in the polyimide and to droplet ejection from the subjacent viscous solution layer. Even though a strong laser source is used, neither the printed material nor the receiver substrate are degraded by optical or thermal effects. In contrast to other LIFT-processes, the ablation products are trapped inside the blister and cannot contaminate the transfer material.

We have recently reported physical structuring of paper-like biosensors by femtosecond laser pulses to establish multi-parameter lateral flow tests [11]. Using the same laser for functionalization by BA-LIFT would, therefore, not require expensive additional equipment for fabricating fully functional multiparameter biosensors.

## 2. Materials and Methods

### 2.1. Laser Setup

**Figure 1:**
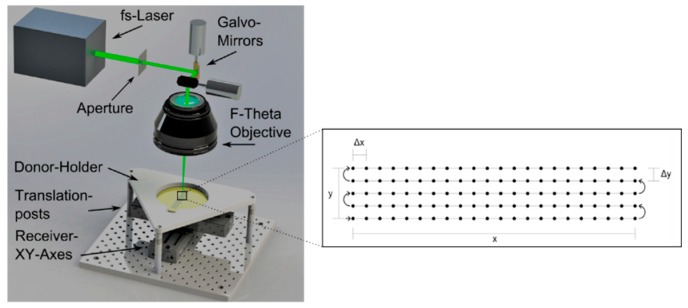
Schematic of the experimental setup and sketch of the typewriter similar receiver movement during the printing process.

The experiments are carried out on a laser micromachining platform (Microstruct C from 3D-Micromac, Chemnitz, Germany) which is equipped with an Yb:KGW femtosecond-laser (Pharos from Light Conversion, Vilnius, Lithuania) with 15 W average power and a minimum pulse duration of 212 fs as has been used for the microstructuring of nitrocellulose membranes in previous works [11]. This laser emits pulses at repetition rates up to 600 kHz at a wavelength of 1030 nm, which can be frequency doubled or tripled to 515 nm and 343 nm. A schematic overview of the experimental setup is shown in Figure 1. The beam is focused on the sample by an f-theta lens with a focal length of 100 mm, where the focal spot can be rapidly displaced using a galvo scanner (Scanlab Intelliscan 14 with RTC 5 controller, SCANLAB GmbH, Puchheim, Germany). 

A pulse picker, in essence a Pockels cell, is used to adjust the pulse energy with the applied voltage. This also enables the laser to emit a single pulse with defined energy and duration on request of the positioning system (either the XY-axis or the galvo scanner). All power measurements are carried out with an internal power meter (Gentec UP19K-15S-W5-XT, Gentec Electro-Optics, Inc., Quebec City, QC, Canada). 

**Figure 2:**
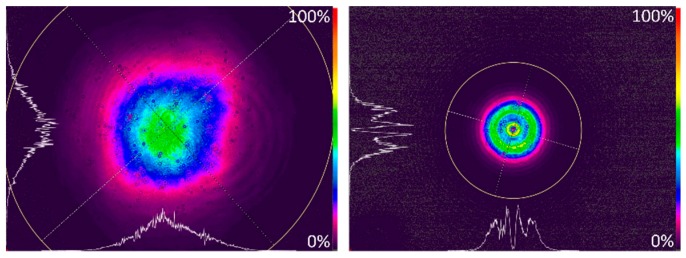
Beam profiler images (recorded with Ophir Spiricon SP928 camera and BeamGage software) of the laser pulses used for the blister-actuated laser-induced forward transfer (BA LIFT) process measured before the focussing optics. Left image: Raw beam with a diameter of 3.6 mm; Right image: “shaped” beam after passing through a 1.5 mm circular aperture. Utilized parameters: Pulse energy *E*_p_ = 2.25 µJ and λ = 515 nm.

In DRL-LIFT processes a flat top beam profile with sharp edges is advantageous because it provides homogeneous irradiation in a well-defined section of the transfer material or the intermediate dynamic release layer [12,13]. In our early experiments, we found that blisters created with a Gaussian beam profile are very fragile, because ablation is not homogeneous and the blister skin in the center is thinner than for the rest of the blister. Similar observations are known from other groups working on BA-LIFT [14]. Therefore, the beam with a Gaussian profile (TEM_00_ M² <= 1.2) and a diameter of 3.6 mm is shaped by passing a circular aperture with 1.5 mm diameter to cut off outer parts of the beam, which almost resembles that of a flat top beam. Thereby, blisters with a wider, but flatter shape, can be fabricated over a broad range of pulse energies. A comparison of both beam profiles is shown in Figure 2.

Special fixtures and positioning systems are required for the precise movement of the donor in relation to the receiver substrate. Especially the gap in between both substrates is of great importance for the quality and reliability of the transfer and has to be precisely controlled. Typical dimensions of the gap for transferring liquid material range from 1 mm down to several µm [15].

For the transfer of individual droplets with no overlap a rather simple setup using spacers or precision-machined mechanical fixtures, which hold the receiver and the donor substrates at a defined distance is sufficient. However, if homogeneous functionalization areas shall be generated by a pattern of very small and overlapping pixels, small droplets have to be ejected at overlapping receiver positions. Since a fresh blister can be created only once at every donor position a special positioning system had to be developed (see Figure 1) and integrated in the laser micro fabrication platform. It consists of two sets of high-speed linear translation stages and drivers (Thorlabs DDSM100/M and K-Cube brushless servo driver, Thorlabs, Inc., Newton, NJ, USA), which are combined to form an additional XY-Axis system for the precise positioning of the receiver substrate against the donor substrate, while keeping a fixed distance. The receiver substrate is fixated on top of the axis system by a circular vacuum chuck with 100 mm diameter made from a porous ceramic material. Fine adjustable posts (Thorlabs) are used for mounting and levelling of the donor substrate holder and for controlling the gap between both substrates.

The XY-axis system which is an integral part of the laser micro fabrication platform can control the position of the donor, but at the same time moves the receiver and is therefore only used for initial positioning and remains stationary for most of the time. The desired position of the focal spot and the resulting blister on the donor film, or rather on the intermediate thin polyimide film, is addressed by the galvo scanner. 

The functionalization pattern is realized by printing during a typewriter similar motion: The scanner optics moves the focus position in horizontal lines, while pulses are emitted at defined intervals to generate blisters in the polyimide layer and to induce local material transfer. The receiver substrates axis system follows the spot movement with the same vector and– the speed difference between both movements is used to adjust the overlap of the transferred droplets. The scanner and the LIFT stage axis systems are synchronized by using addressable IO-Ports that can be controlled from the automation environment.

### 2.2. Donor Preparation

The rigid donor carrier substrate has to be made from a material that shows only minimal absorption at the laser wavelength to prevent ablation or thermal damage (like micro cracks). Ultra-short pulsed laser beams are able to ablate even transparent materials through non-linear absorption processes. As long as the focus position is located at the interface between glass and polyimide the energy density in the glass carrier remains below the threshold for non-linear absorption and no ablation or damage will occur. A 4”-borosilicate glass wafer (Borofloat®, Schott Germany) with a thickness of 700 µm was selected as the carrier substrate and spin coated with 1 mL Spin-On Polyimide (PI-2610, HD-Microsystems, Parlin, NJ, USA). With a rotational speed of 2000 rpm the thickness was adjusted to 3.5 µm. Afterwards, the polyimide layer is exposed to two softbake steps at 90° and 150 °C each for 90 s before a final curing step at 350 °C for 30 min is administered to completely evaporate the solvents. A precise control of these steps is of the utmost importance to ensure consistent mechanical properties, which have a large influence on the blister and droplet formation, and therefore on the reliability of the transfer process. To improve the film thickness repeatability of the donor ink outer parts of the polyimide are peeled off after cutting a central rectangle with dimensions of around 50 mm × 70 mm with a scalpel. 

As a next step the donor ink is applied by means of a profile rod. A small volume of the ink (20–40 µL) is applied in a line on the edge of the rectangular polyimide film and is distributed over the surface by dragging a profile rod with notches of defined depths (ZSA 2110, Zehntner GmbH, Sissach, Switzerland, for nominal wet film thicknesses of 4.57 and 6.86 µm as well as HR01, MTV Messtechnik, Erftstadt, Germany, for 10 µm film thickness) at a constant speed over the polyimide. The composition of the donor ink is crucial for the LIFT transfer. In order to allow a comparison with the results of not blister assisted LIFT approaches from other groups [16] a similar ink composition was selected.

Depending on the type of biosensor different forms of biochemical detection and functionalization can be used. In our work, sandwich immunoassays on nitrocellulose membranes are investigated. The immobilisation of antibodies on nitrocellulose membranes by traditional dispensing methods is a well-known process and used for the commercial production of lateral flow tests (LFT). Due to the high protein binding capacity of the membrane material, the antibodies within a transferred droplet will rapidly bind to the porous structure, while liquids will wick through the membrane and evaporate [17]. For a simple assay the capture antibodies for the detection line and non-specific antibodies for the control line have to be immobilized in a well-defined shape on the membrane. For some specific assays, it can also be necessary to print an additional line with just the antigen in order to compensate for the so-called Hook effect [18], which might lead to false negative tests if antigens are abound in the sample. Both the antibodies and the antigens are usually dissolved in a buffer solution, in most cases phosphate buffered saline (PBS). In normal concentrations they usually have no influence on the rheology of the PBS, which allows for a calibration and optimization of the printing process just with the buffer solution as the transfer material. 

Research on other variants of LIFT uncovered, that the rheology of the donor ink, in particular its viscosity, has a tremendous influence on the quality of the droplet formation and the repeatability of the transfer [19]. A higher viscosity is known to stabilize the fluid jet during transfer, which allows for larger pulse energy windows as well as better-defined droplets [20]. PBS exhibits a cinematic viscosity of 0.8882 centipoise at room temperature, which is too low for a controllable transfer. In addition, thin films of PBS also evaporate quite rapidly, thus severely limiting the time the coated donor system can be used before progressing evaporation influences the quality of the transfer process. To address both of these problems glycerine (Sigma Aldrich) is added to the buffer solution, which is thereby adjusted to a viscosity typical for inkjet inks (8.91 cP for a 1:1 mixture of glycerine and PBS). We evaluated whether this addition will have an impact on the quality of the sensor response by visually comparing the results of LFTs functionalized by manual dispensing with identical antibody concentrations in pure PBS and in a 1:1 mixture of PBS and glycerine. While the definition of the edges of the functionalized area appeared to be a bit worse when adding glycerine, the intensity of the colour change in this area remained similar. 

The cured polyimide layer is hydrophobic, which poses a challenge for the ink coating. Lowering the surface energy of the PI-Layer with an O_2_-plasma treatment was not sufficient for coating with PBS or with a 1:1 glycerine/PBS mixture. To ensure a homogenous coating a surfactant had to be added. In the LIFT investigations of other groups [16,21], sodium dodecyl sulfate (SDS) was added to the donor ink not containing bioactive components. However, SDS is rather powerful and inhibits the non-covalent bonds with and within proteins, thus denaturizing the proteins [22] and should therefore never be used with active proteins. As an alternative Tween 20 was selected, which does not affect protein activity and is already used in several of the blocking and washing solutions for LFTs and its components. While Tween concentrations above 0.05% were beneficial to the homogeneity of the coating process, the immobilization of the antibodies turned out to be severely inhibited. The antibodies appeared to be immobilized on a very small region at the edges of the evaporated manually applied droplet of 0.5 µL, like a coffee-ring stain. In the end a combination of Tween 20 in a concentration of 0.05% together with a plasma treatment of the donor wafer prior to the coating step provided good results.

### 2.3. Parameter Selection

In the basic LIFT process with solid transfer materials process parameters as the laser wavelength, beam profile, pulse duration, focal diameter, fluence or pulse energy, and the gap between donor and receiver substrate are strongly influencing the print result. For blister-actuated laser-induced forward transfer (BA LIFT) with liquid donor material, the number of variables in the system even increases, since the thickness of both the donor and the intermediate polyimide film as well as the viscosity and surface tension of the donor material also have a large influence.

**Figure 3:**
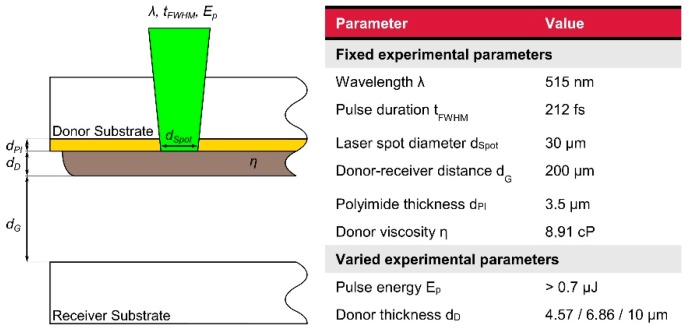
Important parameters of the BA LIFT process.

Despite the enormous number of potential parameter combinations only variations of the laser pulse energy $E_{p}$ as well as the thickness $d_{\mathrm{PI}}$ of the donor material layer were studied and wavelength λ, pulse duration $t_{\mathrm{FWHM}}$., laser spot diameter $d_{\mathrm{spot}}$, viscosity η of the donor material and donor-receiver distance $d_{G}$ were kept constant at values shown in Figure 3. This approach allowed us to avoid optimization with interdependent parameters and to concentrate on the important but experimentally easy to adjust parameters. Parameters, which are essential for the formation of the blisters in the polymide layer, were first studied without adding the liquid donor material layer. Since a femtosecond laser induces multi-photon absorption the formation of blisters in initial experiments was possible with all available wavelengths (1030, 515, and 343 nm). Apart from a variation in the size of the focal areas and in blister formation threshold pulse energies, no fundamental difference could be observed for the different wavelengths. Therefore, only λ= 515 nm was investigated in the parameter study. The positioning system for the independent translation of donor vs. receiver substrate was not activated during these first experiments. Donor wafers coated with spin-on polyimide are placed onto the LIFT stage. The focal plane is determined with a high-accuracy CCD laser triangulation sensor (Keyence LK-G10, Keyence Corporation, Osaka, Japan) before the beginning of the laser process by determining the top surface of the donor substrate and subtracting the thickness of the glass wafer, which gives the interface plane between the glass and the polyimide layer. For each parameter combination, the laser platform is programmed to deliver 25 nominally identical pulses in a 5 × 5 arrangement with a pitch of 200 µm in both x and y-direction. These patterns are repeated in the Y-direction with increasing pulse energy.

The thickness of the polyimide layer was varied by using different spin-on speeds. The donor substrate is flipped and placed into the experimental setups donor holder with the polyimide film facing downwards, away from the laser optics. After laser processing the donor substrate is removed from its holder and the resulting blisters are investigated by laser scanning microscopy (Keyence VK-X260K, Keyence Corporation). 

For the liquid transfer parameter study a borosilicate glass substrate with excellent surface smoothness is used as receiver substrate. It is fixated on the vacuum chuck of the laser system and its surface plane Z-position is determined with the laser displacement sensor. The donor coated with test ink is flipped and positioned over the receiver with the liquid layer facing towards the receiver. The working distance in between both substrates is determined by measuring the Z-position of the donor surface. After laser processing, the receiver substrate is investigated by laser scanning microscopy (Keyence VK-X260K, Keyence Corporation). All measurements are carried out immediately after the transfer to limit the influence of evaporation.

### 2.4. Shadowgraph Setup

In order to gain a better understanding of the mechanics of the transfer process through blister generation and droplet formation, the transfer is visualized using shadowgraph imaging, a technique that has been widely used as a tool for particle flow visualization. It relies on the general principle that a shadow is cast wherever there is a significant optical density gradient in the medium through which light passes [23]. In this way, a shadow image can be captured at the focal plane of a conventional complementary metal-oxide-semiconductor (CMOS)-camera. 

Using a sufficiently fast switching light source this process can be used to essentially freeze and capture the motion of small objects travelling up to 100 mm/s. Xenon flash lamps are often used for this purpose, since they offer pulse lengths in the µs-range, but they are not readily available with higher pulse rates. A pulsed laser source could also be used for the sub-µs burst of light either directly or in an indirect way by stimulating a vial of fluorophore; however, monochromatic light sources are not very well suited for this type of imaging, since they can produce speckle effects on the sensor [24]. With their comparatively low price and short coherence length, high-power LEDs are a viable alternative. They can be driven by current pulses to achieve illumination pulses down to 230 ns duration and their luminosity is very stable while operating in a pulsed mode [25].

**Figure 4:**
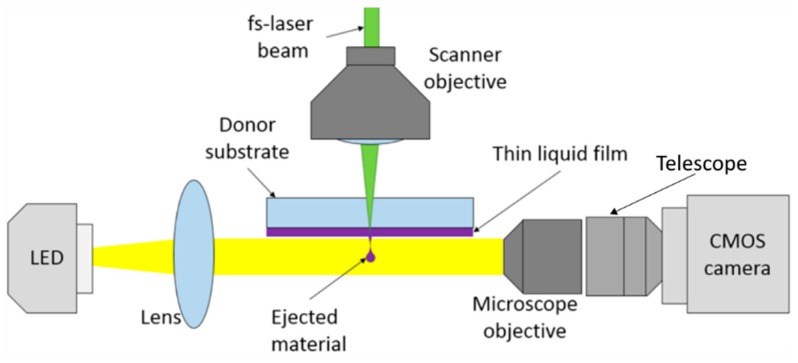
Schematic overview of the shadowgraph setup used for the visualization of the droplet transfer.

A schematic of the utilized shadowgraph setup is shown in Figure 4. A CMOS-Camera (Basler acA2040-90uc, Basler AG, Ahrensburg, Germany) in combination with a 100× magnification microscope objective (Olympus SLMPLN with 7.6 mm working distance, Olympus Corporation, Tokyo, Japan) is used for the imaging. Since the image exiting the aperture of the microscope only has a diameter of around 3 mm a Keplerian telescope is installed between the CMOS-Chip (Sensor size 11.26 mm × 11.26 mm with a resolution of 2040 × 2046 px) and microscope objective. However, due to limited available space, only a twofold magnification can be realized. For illumination, a high-power LED with an average power of 47 W, which achieves a luminosity of 2600 lm at an operating voltage of 35 V (Cree XLamp CXA2520, Cree, Inc., Durham, NC, USA), is mounted on a heat sink. A bi-convex lens is used to direct the light onto the microscope objective and focus on the substrate plane where the transfer should be observed.

The LED is operated in pulsed mode, to provide stroboscopic illumination with durations below 250 ns. In combination with the long off-durations this allows the operation of the LED well beyond its damage threshold for continuous operation [26]. For this purpose, a pulsed LED driver circuit was adapted from Reference [27] with slight modifications and supplied with a 40 V power supply. This circuit consists of a bank of capacitors, which is charged by the power supply, and a combination of a metal-oxide-semiconductor field-effect transistor (MOSFET) power transistor and driver, which can rapidly discharge the capacitors and drive the LED for a short amount of time. The driver can be triggered by an external logic signal to control the onset and duration of the pulses. 

Both the camera and the pulsed light source have to be triggered precisely so that the generation and movement of the droplet gets captured at well-defined moments after the impact of the laser pulse. In contrast to other shadowgraph setups for observing LIFT processes or laser ablation plumes where a multichannel signal generator is used to trigger camera, laser, and light sources, and we used a combination of two of the laser sources internal signals to trigger the light source and the camera. In order to visualize the transfer this automation software was used to request a single laser pulse from the laser source, while subsequently priming a delay generator to check for two internal laser signals, one of which indicates that the external pulse picker is opened, while the second, cyclic signal, occurs when a pulse has been sent from the oscillator into the regenerative amplifier. The combination of both signals indicates that the emission of a laser pulse is imminent.

The control of experiments was realized by means of an FPGA evaluation board (Xilinx Nexys 4, Xilinx, Inc., San Jose, CA, USA), which was programmed as a delay generator with a universal asynchronous receiver-transmitter (UART) serial communication interface. The latter was used to configure the delays externally by a LabVIEW application, which also handled the image acquisition. The FPGA uses an internal clock frequency of 200 MHz—all signals have to by synchronized to this clock signal, which means that, in the worst case, the FPGA will only be able to react to an incident trigger signal after almost a full clock cycle (5 ns).

## 3. Results

### 3.1. Blister Formation in Polyimide-Layers

**Figure 5:**
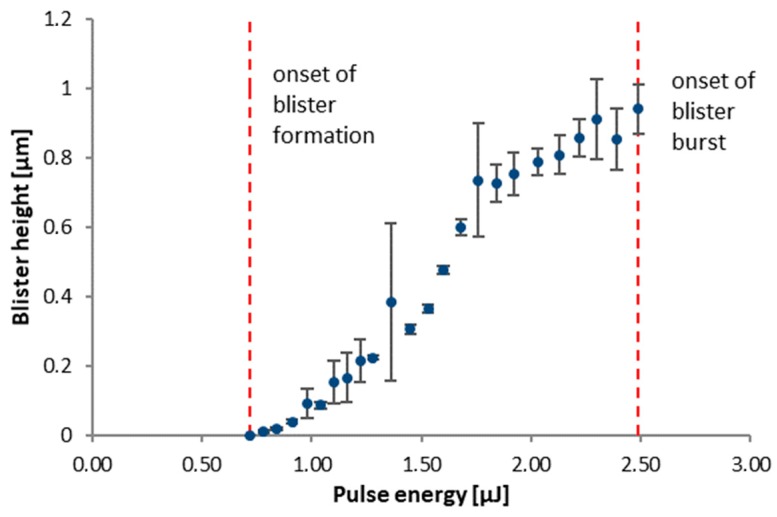
Height of the laser induced blisters in polyimide film with $d_{\mathrm{PI}}$ = 3.5 µm as a function of the pulse energy $E_{p}$ (λ = 515 nm laser wavelength with shaped beam). Error bars indicate standard deviation obtained from 6 measurements.

Arrays of blister patterns were fabricated on donor wafers with varying pulse energies. Thereby obtained blister sizes for a polyimide film thickness $d_{\mathrm{PI}}$ = 3.5 µm are shown in Figure 5. Below a threshold of $E_{p}$ < 0.7 µJ, only slight modifications of the polyimide surface, but no blister formation is observed. With increasing pulse energies, the blister height rapidly increases. Furthermore, an increase of the base diameter of the blister can be observed. At $E_{p}$ > 1.7 µJ, the blister height starts to saturate and blisters fabricated with $E_{p}$ > 2.5 µJ tend to burst. 

While thinner polyimide layers offered a larger pulse energy window for the formation of homogeneous blisters, it was observed that they appear to be quite fragile. A polyimide film thickness $d_{\mathrm{PI}}$ = 3.5 µm, which was adjusted by using a spin-on speed of 2000 rpm, appears as a good compromise between the robustness of the blisters and repeatability of the blister formation. A detailed discussion on the blister formation mechanism is found in literature [28].

### 3.2. BA-LIFT of Test-Ink 

Ink coating was carried out manually by means of profile rods with different groove depths. The resulting thickness was determined by laser scanning microscopy at multiple positions on the wafer. The results of these measurements are shown in Table 1. As expected, the thickness of the liquid films was lower than average on the edges of the film and higher in the middle of the wafer (fluctuations of around ± 10%). 

**Figure 6:**
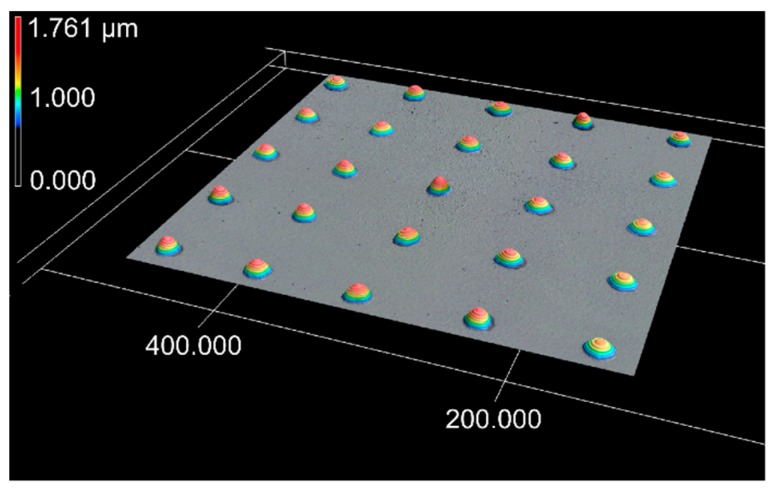
3D-LSM image of a 5 × 5 array of ink droplets transferred by BA-LIFT onto a glass substrate. Z-axis is scaled by a factor of 10 compared to x and y axes. Process parameters: λ = 515 nm wavelength and $E_{p}=$ 1.75 µJ, gap between donor and receiver $d_{G}$ = 200 µm, film thicknesses $d_{D}$ = 1.55 µm liquid donor, and $d_{\mathrm{PI}}$ = 3.5 µm polyimide.

For studying of the transferred volumes glass acceptor substrates were used. An example of the 5 × 5 liquid transfer array on a glass substrate is shown in Figure 6. The volume of very small satellites around the main droplets can be considered negligible.

**Figure 7:**
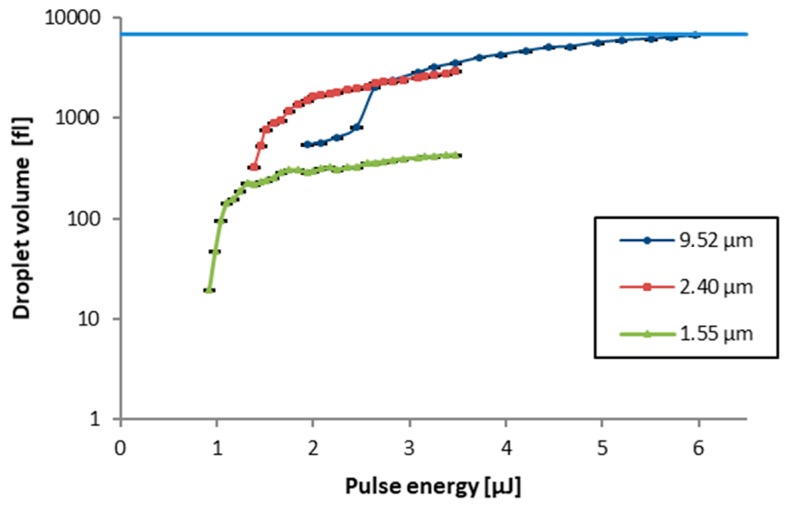
Volume of the transferred droplets as a function of the pulse energy $E_{p}$ of the inducing laser pulse for 3 different liquid donor film thicknesses $d_{\mathrm{PI}}$. Process parameters: λ = 515 nm, gap between donor and receiver $d_{G}$ = 200 µm, and polyimide film thickness $d_{\mathrm{PI}}$ = 3.5 µm. Data points are connected by lines to guide the eye. The horizontal line indicates the maximum volume that could be transferred which correlates with cylinder volume with a diameter of 30 µm (laser beam diameter) and the maximum investigated film thickness (9.52 µm).

For each investigated parameter combination, a 5 × 5 array of droplets is transferred and droplet volume mean values as well as standard deviations are calculated. Figure 7 shows a clear increase of the droplet volume with raising pulse energy $E_{p}$, which also means the volume of the ejected droplet correlates with the size of the laser-induced blister. A pulse energy threshold, below which no droplet transfer occurs, is observed: 0.92 µJ for the 1.55 µm (which is close to the blister formation threshold of 0.7 µJ) to 1.9 µJ for the 9.52 µm donor film. For thicker liquid films this threshold is further exceeding the threshold observed for blister formation without liquid film (Figure 5). This can be attributed to the amount of increasing surface energy that has to be overcome to form a liquid jet by the transfer impulse of the emerging blister as well as the increasing kinetic energy of the accelerated donor material. 

Furthermore, the thickness of the donor material film has a large influence on the droplet volume. With the thinnest investigated donor film ($d_{\mathrm{PI}}$ = 1.55 µm), droplet volumes in the fL-range were measured up to a volume of 430 fL at the end of the investigated pulse energy range. The somewhat thicker ($d_{\mathrm{PI}}$ = 2.40 µm) film drastically increased the transfer volume to up to 3.0 pL toward the end of the investigated $E_{p}$ range. Using these two donor film thicknesses, a highly repeatable transfer with a low standard deviation in transferred volume of below 0.2% is obtained. 

The investigation of the thinner (1.55 µm and 2.40 µm) donor material layers was limited to a maximum pulse energy value of 3.48 µJ since larger pulse energies increased the probability that the blister would burst, which limits the reliability of the transfer. However, the laser-induced blisters appeared to be more stable against bursting when a much thicker donor film ($d_{\mathrm{PI}}$ = 9.52 µm) is applied, probably because the blister formation is more damped by a thicker donor film. Even though material transfer can be observed with pulse energies between 2.0 and 2.5 µJ large volume fluctuations limit the usability in this range. A possible explanation for these fluctuations is the increasing influences of fluid behavior in the thicker donor film influencing the emerging blister. However, a stable transfer with better reproducibility occurs for higher values of $E_{P}$ between 2.5 and 6 µJ with droplet volumes between 2.0 and 6.6 pL.

A saturation can be observed in the transfer volume curves for all the investigated film thicknesses, which is a result of the limited amount of liquid available on top of the emerging blisters. For example, a cylinder with a diameter of 30 µm (as measured for the blister base area diameter) and a height of 9.52 µm (the measured film thickness for the thickest investigated donor material layer) has a volume of 6.722 pL, which is in very good agreement with the saturation value (see blue horizontal line in Figure 7). This is a simplifying consideration, because in reality some liquid will also flow from around the blister towards its center during jet formation. However, it indicates that, only with a broader laser spot, more material could have been transferred.

**Figure 8:**
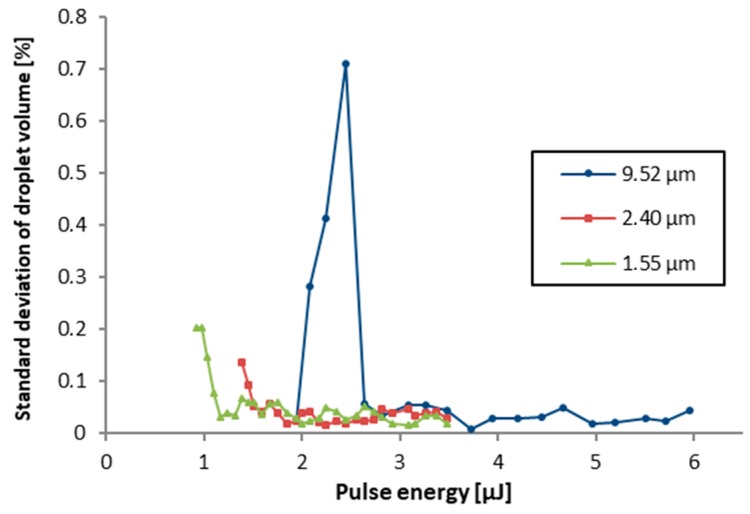
Standard deviation obtained from measured droplet volumes of 5 × 5 arrays at three different fluid film thicknesses as a function of the pulse energy. Process parameters: λ = 515 nm, gap between donor and receiver $d_{G}$ = 200 µm and polyimide film thickness $d_{\mathrm{PI}}$ = 3.5 µm. Data points were connected by lines to guide the eye.

The volume standard deviations are shown in Figure 8. It can be observed that the reproducibility of the transfer is lower for pulse energies closer to the transfer threshold for the respective film thickness, but drops below 0.05% for higher pulse energies, which indicates very good reproducibility. Only for the thickest investigated donor layer a much less reproducible transfer is obtained between 2.0 and 2.5 µJ, which might indicate a process regime, which is more influenced by flow within the liquid layer. The interfaces (capillary effects) are not scaling with the film thickness, however the freedom of viscous convection increases. At higher energies, the liquid layer starts to be more completely affected by the blister impulse, and finally, the complete volume above the blister base area is ejected (see also Figure 7).

These results clearly show that the investigated BA-LIFT process enables a very reliable transfer of very small volumes of liquid (volume fluctuations of less than 0.05% for higher pulse energies) combined with the high positioning accuracy of the laser platforms stage (±5 µm). In fact, the smallest demonstrated droplet volume of less than 20 fL when using a liquid film thickness of 1.55 µm is far below what any other solution printing or micro arraying processes for biosensor functionalization have ever achieved. However, a continuous variation of the droplet volume by using different pulse energies is only feasible within a limited range, which depends on the thickness of the liquid donor film, while this might be viewed as a limitation of the process it is shared by most of the alternative approaches. The volume of inkjet and contact printed droplets is also limited by the dimension of the nozzle or contact pin. In the case of the presented BA-LIFT process the transition to a larger droplet range can be rapidly realized by applying a thicker donor film without the exchange of mechanical equipment and subsequent re-calibration. 

### 3.3. Transfer Visualization by Shadowgraphy

**Figure 9:**
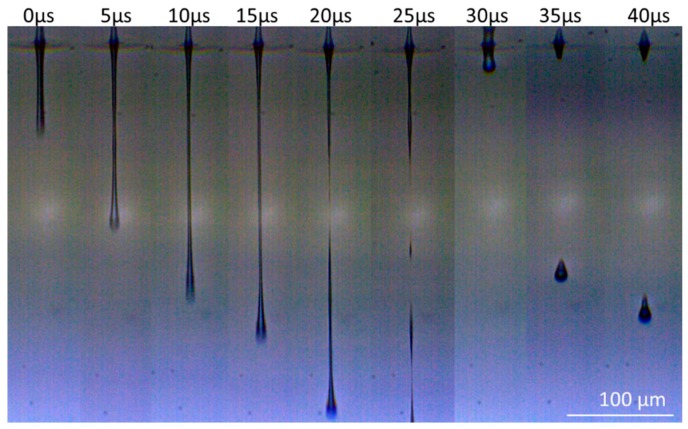
A stroboscopic sequence of shadowgraph images showing the droplet ejection dynamics at pulse energy $E_{p}$ = 2.5 µJ, process parameters: λ = 515 nm, film thicknesses $d_{D}$ = 9.52 µm liquid donor, and $d_{\mathrm{PI}}$ = 3.5 µm polyimide.

The stroboscopic approach only allows capturing a single image per transfer. The sequence displayed in Figure 9 therefore consists of shadowgraph images of different droplets, which were transferred with identical parameters. The time *t* = 0 is defined as the moment when the earliest image is recorded after the electrical trigger is received from the laser. A systematic delay in the µs range between the laser pulse and the earliest image (also adding to the timing of further images) leads to a start of jet formation already before *t* = 0, as can be seen in Figure 9. This delay can be attributed to the discharge of the capacitors powering the LED. 

It can be observed that—in the absence of a receiver substrate, which would normally be situated at 200 µm distance to the donor—a continuous jet is ejected from the donor substrate after the blister has formed. The expansion speed was estimated from shadowgraph images to be around 18 m/s. After about 20 µs the jet collapses, and most of its volume continues to move in the same direction, while a fraction of the liquid stream merges again with the donor film, which causes a second ejection in the form of a larger droplet after 30 µs. The ejection in presence of a receiver substrate could not be observed, since the gap between both substrates (200 µm) is too narrow for allowing shadowgraphy imaging. Nevertheless, the volume of the first emission in form of a jet (estimated based on the shadowgraph images to be about 1 pL) together with the second emission in the form of an elongated droplet (also about 1 pL) is in agreement with the measured volumes transferred to glass for these laser parameters (around 2 pL). Furthermore, the observed ejection behavior is comparable to the investigated experiments and the developed computational model in [14]. Therefore, we can assume that a continuous jet is formed during the transfer process, which bridges the gap between both substrates. After the collapse of the first jet, one or more additional droplets are formed and transferred to the receiver, where they contribute to the transferred volume. 

## 4. Proof-of-Concept: BA-LIFT Functionalized Paper-Like Biosensor

The feasibility of BA-LIFT printing for biosensor functionalization is evaluated by using a bio-ink with detection antibodies for a quantitative CRP-Assay in combination with nitrocellulose membranes as receiver substrates, which was structured before on the same laser platform to establish channel structures. Structuring allows to guide the test liquid from a common sample application pad through multiple, parallel channels with no cross-contamination into a shared absorbent pad. This can abolish restrictions of conventional non-structured lateral flow tests, most importantly the inability to get more than a semi-quantifiable result and the limitation to a single parameter per test. Details on the laser structuring process for the rather delicate and flammable membrane material have been reported earlier [11]. Each of the four 1 mm wide parallel channels is equipped with three reaction zones with a diameter of 1.5 mm, which have to be functionalized with reaction chemicals, e.g., detection/control antibodies, in order to create an immunoassay. Furthermore, the test strip is equipped with alignment markers for easy and robust readout with smartphones and a barcode for test identification, while maintaining a footprint comparable to commercially available devices. The test platform consists of off-the-shelf materials for lateral flow tests: Sartorius Unisart CN 140 (Sartorius AG, Göttingen, Germany) was used as the laser structured nitrocellulose membrane strip, while cellulose fibers were selected as sample and absorbent pad (Merck Milipore C083, Merck Millipore, Burlington, MA, USA) and fiberglass as the material for the conjugate pad (Merck Millipore G041).

While other assays have recently been developed for this laser structured multiparametric later flow test using conventional dispensing methods for the bio-ink [29], for this proof of concept, we selected a simple colorimetric assay for the detection of c-reactive protein (CRP) using antibodies labelled with colloidal gold, since it is quite easy to establish and doesn’t require heightened safety measures during handling. The labelling process was carried out using the same labelling protocol as described in [29]. The CRP-assay normally uses three subsequent detection zones/lines functionalized with different antibody/antigen solutions: For the detection line we used monoclonal antibodies to human C-reactive Protein (stored with a concentration of 1 mg/mL) purchased from Dunn Labortechnik (Thelenberg 6, 53567 Asbach, Germany) while polyclonal Goat anti Mouse IgG Antibodies (AbD Serotec® obtained from Bio-Rad AbD Serotec GmbH, Zeppelinstr. 4, 82178 Puchheim, Germany) diluted to a concentration of 0.24 mg/mL is used as a control line. An additional antigen-line in between detection and control lines to recognize the aforementioned Hooke-effect was omitted since only test liquids with known concentrations were used.

The fiberglass conjugate pad is structured into a comb-structure by means of a laser cutter to enable a more focused flow of the test liquid and subsequently blocked with a mixture of 10 mL PBS, 50 mg BSA und 25 μL Tween® 20 (for a batch of 20 pads). The pads are covered with this mixture and dried overnight at 37 °C. The conjugate itself, antibodies to human CRP labeled with colloidal gold nanoparticles in a PBS buffer with 10% sucrose, is manually applied to each pad and dried overnight at 37 °C as well, 15 µL per pad were used. The sucrose is necessary to encapsulate the antibodies during this drying step and prevents a bonding to the conjugate pad. As soon as the test liquid is applied the sucrose matrix, it dissolves and the antibodies mix into the test liquid. 

Since only a fraction of the antibodies in a shallow layer below the membrane surface can be recognized by low cost optical readout a rather large amount of antibody solution is required for each reaction zone. For comparison of the printing process to other functionalization methods a reference zone (a second antigen detection), as well as a control zone, which is used to capture labeled conjugate-antibodies to verify the viability of the conjugate, are functionalized by manual spotting. The reference zone is spotted with a 0.2 µL droplet of the carrier-ink developed for the BA-LIFT process loaded with the capture antibody, while the control zone is spotted with 0.2 µL of the IgG-antibody solution (0.24 mg/mL). 

Manual spotting using Eppendorf pipettes is very unreliable regarding the positioning accuracy and from a large single droplet a lot of the chemicals will wick vertically beneath the surface of the membrane. Therefore, only a smaller amount of reaction chemicals will be required with BA-LIFT printing. A volume of 0.1 µL was considered to be sufficient for the BA-LIFT process. Based on the results shown in Figure 7 for the 10 µm donor layer a pulse energy of 4.84 µJ was selected, which enables the transfer of 5.0 pL per droplet transfer event—this means that 20000 droplets are required per reaction zone. Without relative movement between the donor and receiver substrate the pitch between the blister positions cannot be smaller than 50 µm to prevent merging blisters and only 20 × 20 droplets, in total 2 nL, can be transferred in a 1 mm × 1 mm rectangular functionalization structure. However, with the use of the LIFT stage and typewriter similar printing described in Section 2: the detection zones were functionalized in a line-pattern with a line width of 1.2 mm and a length of 0.2 mm. The nitrocellulose membrane is moved in x-direction while the laser scanner moves the focus position in the same direction but with a higher speed and the donor substrate remains stationary. The functionalization pattern consists of 20 lines of 1200 droplets with a pitch of 1 µm (x direction). The line pitch was set to 10 µm (y direction). On the donor substrate, the pitch between blisters was set to 58.8 µm in x and 40 µm in y-direction. Thereby, in total, 24000 droplets were transferred per functionalization pattern, which corresponds to 120 nL per recognition zone.

**Figure 10:**
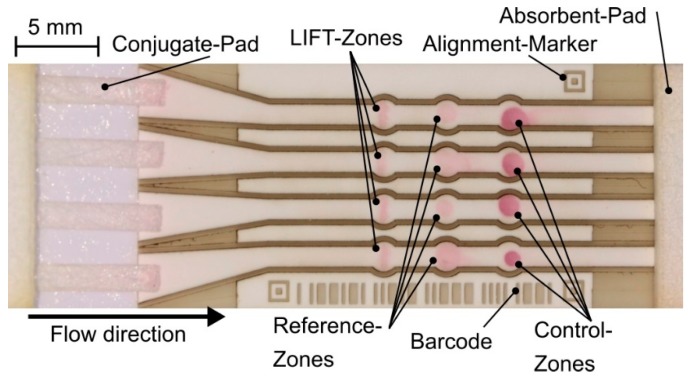
Prototype of fully laser fabricated multichannel lateral flow test. The laser structured nitrocellulose membrane consists of 4 parallel channels with 3 reaction zones each. For the proof of concept with CRP detection the first zone of each channel is functionalized with 120 nL of capture antibody solution (24,000 droplets with an individual volume of 5 pL) by BA-LIFT, while the other zones, which appear far less precisely defined, are manually spotted.

After the BA-LIFT transfer and manual functionalization of the other zones the membrane is dried for 1 h at 37 °C. Other components as sample application pad, conjugate and absorbent pads are mounted with a small overlap to the membrane on the LFT-backing card. As test liquid 250 µL of CRP-solution (25 mg/mL) is applied to the application pad. After 5 min, the test result can be observed, as shown in Figure 10. 

All zones functionalized with the BA-LIFT process exhibit a localized and well defined homogeneous color change free of coffee-ring patterns. As expected, the manually spotted zones show large fluctuations and a similar or weaker color change (in the reference zones), even though the spotted volume was almost twice as large as the transferred volume per LIFT-zone. The BA-LIFT appears to most suit the functionalization of biosensor surfaces, even though the full potential of this technique would become apparent when even smaller reaction-zones in tests with even more parameters would be required.

## 5. Conclusions

We presented an innovative flexible digital printing process for the transfer of individual overlapping droplets of bio-ink for the area-selective surface functionalization of multichannel biosensors using the BA-LIFT. This process uses single laser pulses focused through a transparent carrier substrate onto an intermediate polyimide layer to create blisters whose rapid emergence creates the transfer impulse for the ejection of defined droplet volumes from the subjacent layer of bio-ink.

Using bio-ink layers with thicknesses ranging from 4.7 µm to 10 µm this process was able to reliably create and transfer individual droplets with minimal volumes in the femtoliter range, which is much smaller than for comparable methods, like inkjet or contact printing. For functionalization in well-defined reaction zones the printing of patterns with overlapping droplets by means of a specially developed LIFT stage was realized, which enables a relative movement of donor and receiver substrate, while keeping a well-defined spacing between them. As a proof-of-concept these functionalization patterns were used to create reaction zones in a multiparametric LFT based on laser-structured nitrocellulose membrane material. Rectangular functionalization areas of 1.2 mm × 0.2 mm were homogeneously filled with 24000 droplets resulting in a bio ink transfer of 120 nL. The biochemical activity of the transferred CRP antibodies obtained as a color change appeared to be comparable to those in functionalization areas manually spotted with twice the volume. With the extremely small droplets in BA-LIFT, a much higher lateral resolution and better-defined functionalization area is obtained. A coffee-ring pattern which is typical for transfer of the volume in one step, was not observable and the more homogeneous color changes in the detection zones of the biosensor will be beneficial for any optical readout. With the low amounts of buffer solution, the penetration depth into the nitrocellulose membrane is reduced due to faster drying. The expected positive effect on optical sensing in which only a shallow layer of nitrocellulose membrane is reached will be investigated in further research. 

While we successfully demonstrated the feasibility of the BA-LIFT process for the surface functionalization of a LFT with sensing areas in the range of millimetres, the full potential of this technique will become apparent when, in the future, even smaller reaction-zones in tests with even more parameters will be required. 

We are confident that the extremely small and highly reproducible droplet size is of great interest for many other biosensor applications in particular for DNA microarrays, which require only small transfer volumes in the picoliter range.

## Figures and Tables

**Table 1:** Measured average liquid film thicknesses on the polyimde film obtained with different profile rod grove depths.

Grove Depth [µm]	Average Film Thickness [µm]
4.57	1.55
6.86	2.40
10	9.52
